# Supplementary material for: High-Resolution Photoemission Study of Neutron-Induced Defects in Amorphous Hydrogenated Silicon Devices
Source: Nanomaterials (Basel). 2022 Oct 4;12(19):3466. doi: 10.3390/nano12193466 (PMC9565300; doi:10.3390/nano12193466)
Supplement: Supplementary file 1 [file nanomaterials-12-03466-s001.zip › nanomaterials-1888880-supplementary.pdf]

Supporting Information

Article

High-Resolution Photoemission Study of Neutron-Induced Defects in Amorphous Hydrogenated Silicon Devices

Francesca Peverini [1,2,*], Marco Bizzarri [1,2], Maurizio Boscardin [3,4], Lucio Calcagnile [5], Mirco Caprai [1], Anna Paola Caricato [6], Giuseppe Antonio Pablo Cirrone [7], Michele Crivellari [4], Giacomo Cuttone [7], Sylvain Dunand [8], Livio Fanò [1,2], Benedetta Gianfelici [1,2], Omar Hammad [4], Maria Ionica [1], Keida Kanxheri [1], Matthew Large [9], Giuseppe Maruccio [6], Mauro Menichelli [1], Anna Grazia Monteduro [6], Francesco Moscatelli [1,10], Arianna Morozzi [1], Stefania Pallotta [11], Andrea Papi [1], Daniele Passeri [1,12], Marco Petasecca [9], Giada Petringa [7], Igor Pis [13], Gianluca Quarta [5], Silvia Rizzato [6], Alessandro Rossi [1,2], Giulia Rossi [1], Andrea Scorzoni [1,12], Cristian Soncini [10], Leonello Servoli [1], Silvia Tacchi [10], Cinzia Talamonti [11], Giovanni Verzellesi 3[,14], Nicolas Wyrsch [8], Nicola Zema [15] and Maddalena Pedio [1,16]

[1] INFN (Istituto Nazionale di Fisica Nucleare), Sez. di Perugia, via Pascoli s.n.c., 06123 Perugia, Italy
[2] Dipartimento di Fisica e Geologia, Dell'Università degli Studi di Perugia, via Pascoli s.n.c., 06123 Perugia, Italy
[3] INFN (Istituto Nazionale di Fisica Nucleare), TIPFA Via Sommarive 14, 38123 Trento, Italy
[4] Fondazione Bruno Kessler, Via Sommarive 18, 38123 Trento, Italy
[5] CEDAD-Centro di Fisica Applicata, Datazione e Diagnostica, Dipartimento di Matematica e Fisica "Ennio de Giorgi", Università del Salento e INFN-Sezione di Lecce, 73100 Lecce, Italy
[6] INFN (Istituto Nazionale di Fisica Nucleare) and Dipartimento di Fisica e Matematica dell'Università del Salento, Via per Arnesano, 73100 Lecce, Italy
[7] INFN (Istituto Nazionale di Fisica Nucleare) Laboratori Nazionali del Sud, Via S.Sofia 62, 95123 Catania, Italy
[8] Ecole Polytechnique Fédérale de Lausanne (EPFL), Institute of Electrical and Microengineering (IME), Rue de la Maladière 71b, 2000 Neuchâtel, Switzerland
[9] Centre for Medical Radiation Physics, University of Wollongong, Northfields Ave, Wollongong, NSW 2522, Australia
[10] Istituto Officina dei Materiali-CNR, Basovizza SS-14, km 163.5, 34012 Trieste, Italy
[11] INFN (Istituto Nazionale di Fisica Nucleare) and Dipartimento di Fisica Scienze Biomediche sperimentali e Cliniche "Mario Serio", Viale Morgagni 50, 50135 Firenze, Italy
[12] Dipartimento di Ingegneria, Dell'Università Degli Studi di Perugia, via G.Duranti, 06125 Perugia, Italy
[13] IOM-CNR, Istituto Officina dei Materiali, AREA Science Park Basovizza, 34149 Trieste, Italy
[14] Dipartimento di Scienze e Metodi dell'Ingegneria, Università di Modena e Reggio Emilia, Via Amendola 2, 42122 Reggio Emilia, Italy
[15] Istituto di Struttura della Materia-CNR, Via Fosso del Cavaliere 100, 00133 Roma, Italy
[16] Istituto Officina dei Materiali-CNR, V. A. Pascoli s.n.c., 06123 Perugia, Italy
* Correspondence: francesca.peverini@studenti.unipg.it

SI 1 Inelastic Mean Free Path

The inelastic mean free path IMFP may be calculated semi-empirically by the Tanuma-Powell-Penn equation (TPP-2M) [1], which depends on the material molecular mass (*M*), band gap (*Eg),*

density (ρ), and the number of free valence electrons (*NV*). IMFP predictions using TPP-2M are accurate to within 10%.

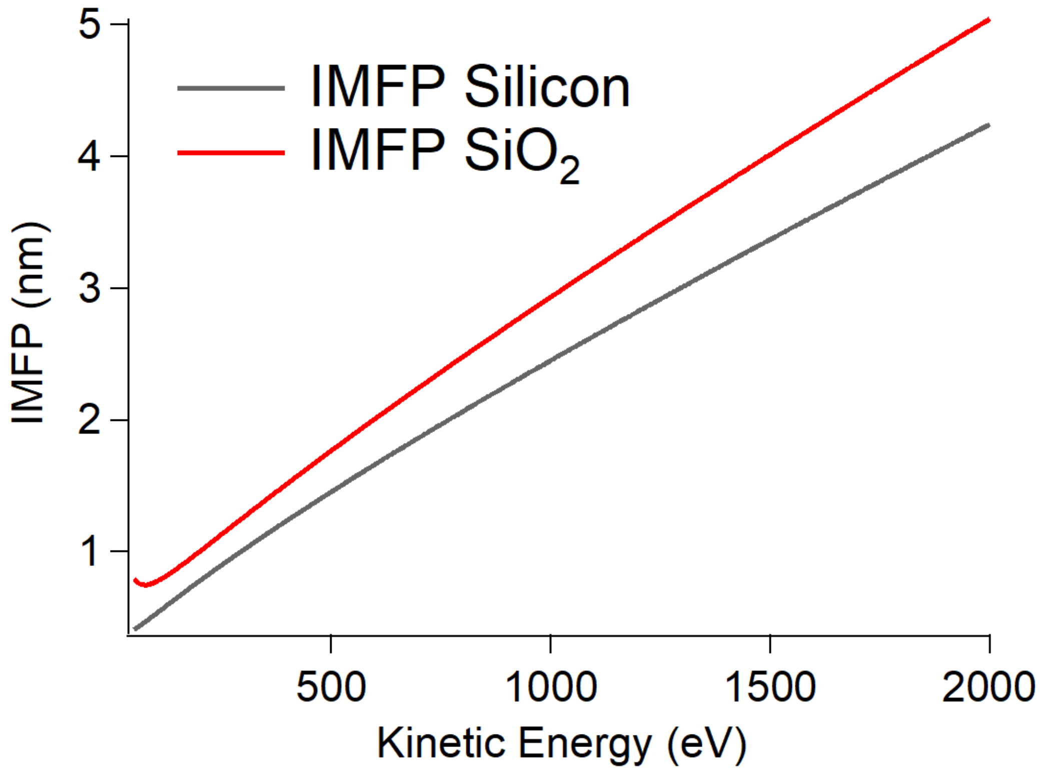

Figure S1 *The IMFP for Si 2p photoelectrons in Si and SiO2 as a function of Kinetic Energy.*

SI 2 Comparison of XAS detection modes

In the Si L2,3 XAS edges the different sampling depths of Fluorescence Yield and the Total Electron Yield detection modes allow for discriminating the contribution of the spectra related to the coating oxide (above 104 eV) to the underneath crystalline silicon.

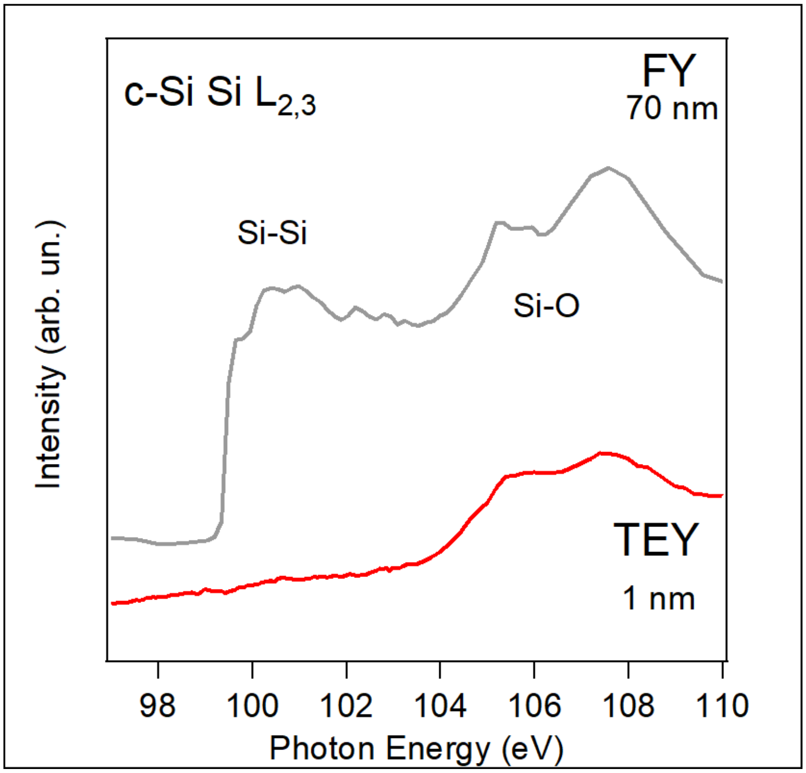

Figure S2 Comparison of Si L2,3 XAS edge taken in Fluorescence Yield (FY) and Total Electron Yield (TEY). The sample is crystalline Silicon with the layer of native oxide of about 3nm.

SI 3 Si 2p core level deconvolution

The Si 2p spectra were deconvoluted into chemically-shifted components using Linear for hv=700 eV and linear or Shirley background for 1400 eV, Voigt doublets with spin-orbit splitting 0.6 eV, $2p_{3/2}$-$2p_{1/2}$ intensity ratio of 1.5, Lorentzian width 100-150 meV, and Gaussian width $\geq$ experimental resolution.

Table S1 Example of fit parameters used for the global fit of the Si2p core level taken at 700 eV and 1400 eV for No irradiated spectra and 1400 eV for Irradiated and annealed samples. For the component of the non-stoichiometric Silicon oxides components see ref.2

		**No Irradiated hv=700**	**No Irradiated hv=1400 eV**	**Irradiated**	**Annealed**
**Component**	**Binding Energy Si $2p_{3/2}$ (eV)**	**Gaussian Width**	**Gaussian Width**	**Gaussian Width**	**Gaussian Width**
c-Silicon (c Si-Si)	99.4	0.35	0.75	0.7	0.75

a-Si&Dangling Bonds (Dis Si)	99.1	0.31	0.85	0.75	0.8
Si-H	99.7	0.36	0.7	0.75	0.75
Si-C	100.1	0.35	0.7	0.7	0.8
SiOx	102.-100.4	1.9	-	1.9	1.7
SiO2	103.5	2.1	2.4	2.0	1.8

SI-4 Effect of sputtering on the Si2p XPS

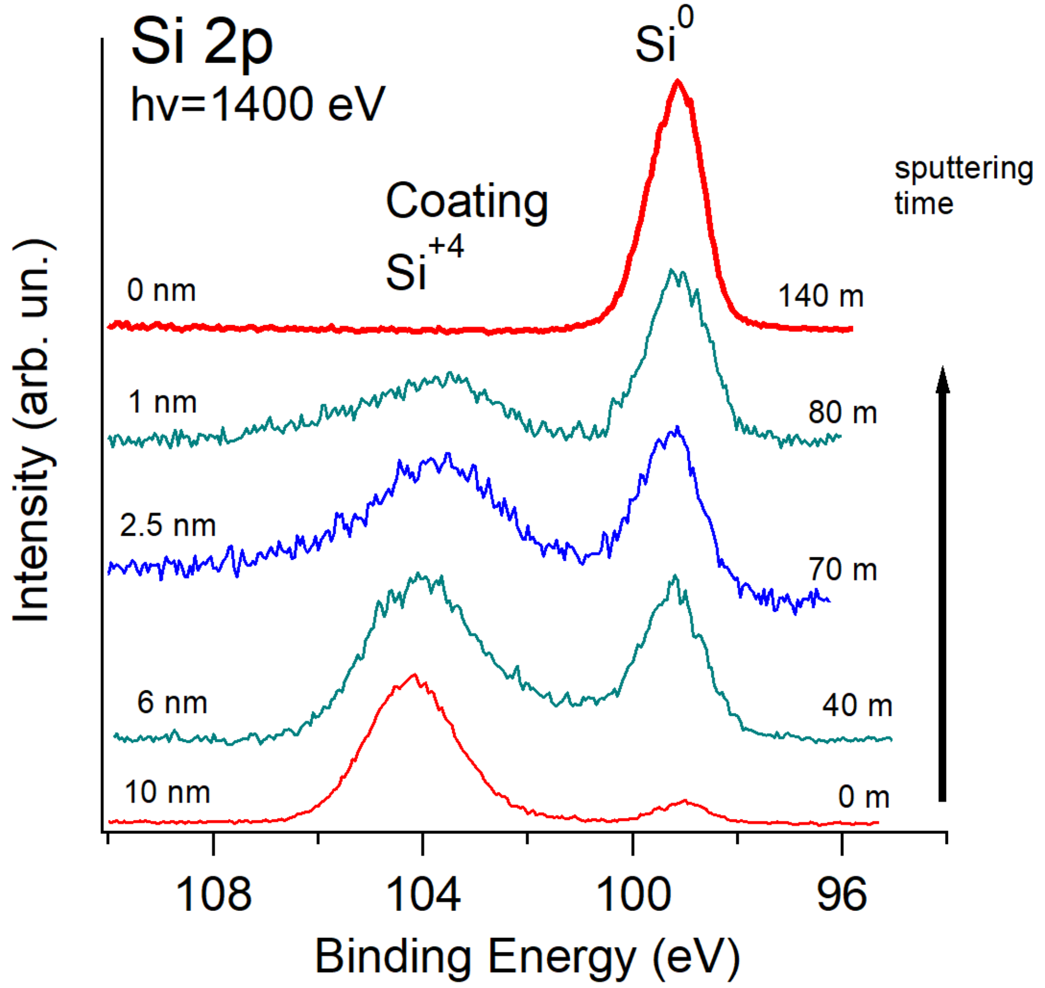

Figure S3 Example of Si 2p evolution as a function of the sputtering time of a a-Si:H reference film, indicating the disappearance of the coating contribution after 140 minutes.

References

1) S. Tanuma, C. J. Powell, and D. R. Penn, "Calculation of electron inelastic mean free paths (IMFPs) VII. Reliability of the TPP-2M IMFP predictive equation" Surf. Interface Anal. 35, 268 (2003)

2) F. J. Himpsel, F. R. McFeely, A. Taleb-Ibrahimi, J. A. Yarmoff, G. Hollinger "Microscopic structure of the SiO2/Si interface" Phys- Rev. B 38, 6084 (1988).
